# Periodontal Disease‐Like Manifestations in Patients Having Acute Leukemia With Normal Complete Blood Counts

**DOI:** 10.1155/crid/6081296

**Published:** 2026-07-03

**Authors:** Jingmei Yang, Wenwen Chen, Panpan Wang, Jiajia Liu, Yi Xu

**Affiliations:** ^1^ State Key Laboratory of Oral Diseases & National Center for Stomatology & National Clinical Research Center for Oral Diseases & Department of Periodontics, West China Hospital of Stomatology, Sichuan University, Chengdu, Sichuan, China, scu.edu.cn; ^2^ Department of Periodontology, Guanghua School and Hospital of Stomatology, Sun Yat-sen University, Guangzhou, Guangdong, China, sysu.edu.cn; ^3^ Department of Pediatric Dentistry, West China Second University Hospital, Sichuan University, Chengdu, Sichuan, China, scu.edu.cn; ^4^ Key Laboratory of Birth Defects and Related Diseases of Women and Children, Sichuan University, Chengdu, Sichuan, China, scu.edu.cn

**Keywords:** acute leukemia, case report, gingival hypertrophy, oral manifestations, osteolytic destruction

## Abstract

Acute leukemia (AL) is a malignant hematopoietic disease with a high fatality rate, and early detection and diagnosis are crucial for subsequent treatment. Clinically, oral manifestations, particularly the ones similar to those of periodontal disease, may be the first signs of AL. The present study reports two patients with complaints of gingival swelling and loosening of teeth, who had normal routine blood clinical tests. After bone marrow puncture, the patients were diagnosed with AL. Despite the blood tests being normal, abnormal gingival manifestations and unexplained looseness of teeth should be treated with suspicion, and AL should be considered a possible differential diagnosis.

## 1. Introduction

Various systemic disorders present with early signs in the oral cavity. Among these, acute leukemia (AL) is frequently accompanied by oral complications [1]. AL is a malignant clonal disease of hematopoietic stem cells, with normal hematopoietic function suppressed at the onset. Globally, leukemia ranked 13th in incidence and 10th in mortality among all cancers in 2022 [2]. AL progresses rapidly, and the average survival period is only approximately 3 months. Without treatment, death can occur even within a few days of diagnosis. Therefore, early detection and diagnosis are crucial for the subsequent treatment and prognosis.

Oral manifestations of AL arising from underlying thrombocytopenia, neutropenia, impaired granulocyte function, or direct leukemic infiltration may precede systemic syndromes and blood disorders. Clinically, oral symptoms such as gingival hyperplasia, gingival swelling, gingival pain, and mucosal ulceration are easily detected, and patients with leukemia often visit the Department of Dentistry first. As for some unusual oral manifestations, complete blood counts are regularly utilized to assist with clinical diagnosis and differentiation from other hematological diseases. However, owing to the rapid progression of AL, some patients present with early symptoms resembling periodontal diseases, albeit with normal complete blood counts, leading to potential misdiagnoses or missed diagnoses. We herein report two cases of AL presenting with gingival symptoms and normal hematological test results, aiming to provide a reference for dentists to identify any possible initial evidence of AL.

## 2. Case Presentation

### 2.1. Case 1

A 45‐year‐old male patient consulted a dentist at the Department of Periodontics, the West China Hospital of Stomatology, Sichuan University, complaining of gingival swelling for more than 3 months. Two months ago, this patient sought medical attention at a local primary hospital because of “gum swelling and spontaneous bleeding.” In light of the patient′s negative history of systemic disease and normal findings on routine blood examination (Table 1) and coagulation function assessment (Table 2), a diagnosis of periodontitis was rendered. The patient underwent supragingival and subgingival scaling, which provided slight symptom relief of gingival bleeding. The treatment lasted for 2 months, but the gingival enlargement did not improve. Given the patient′s unremarkable medical history and normal hematological tests, the dentist simply attributed the gingival swelling to periodontitis without considering other possible diseases, and no further diagnostic examinations were performed.

**Table 1:** Local hospital complete blood count (CBC) result of Case 1.

Test name	Result	Unit	Reference range
White blood cells (WBCs)	5.06	×10^9^/L	3.5–9.5
Red blood cells (RBCs)	5.68	×10^12^/L	4.3–5.8
Hemoglobin (HGB)	174.00	g/L	130–175
Hematocrit (HCT)	50.20↑	%	40–50
Mean corpuscular volume (MCV)	88.50	fL	82–100
Mean corpuscular hemoglobin (MCH)	30.70	pg	27–34
Red blood cell distribution width standard deviation (RDW‐SD)	42.80	fL	37.1–49.2
Red blood cell distribution width coefficient of variation (RDW‐CV)	13.00	%	12–14.3
Mean corpuscular hemoglobin concentration (MCHC)	347.00	g/L	316–354
Platelets (PLTs)	193.00	×10^9^/L	125–350
Platelet packed cell volume (PCT)	0.222	%	0.108–0.282
Mean platelet volume (MPV)	11.5	fL	9.1–12.1
Platelet larger cell ratio (P‐LCR)	35.90	%	11–45
Platelet distribution width (PDW)	17.1↑	fL	9.9–16.1
Platelet larger cell (P‐LCC)	69.00	fL	30–90
Neutrophils (NEUT%)	65.50	%	40–75
Lymphocytes (LYMPH%)	26.10	%	20–50
Monocytes (MONO%)	5.6	%	3–10
Eosinophils (EO%)	2.10	%	0.4–8
Basophils (BASO%)	0.7	%	≤ 1
Neutrophils (*n*) (NEUT#)	7.44↑	×10^9^/L	1.6–6.8
Lymphocytes (*n*) (LYMPH#)	2.95	×10^9^/L	0.64–3.88
Monocytes (*n*) (MONO#)	0.920↑	×10^9^/L	0.096–0.776
Eosinophils (*n*) (EO#)	0.150	×10^9^/L	0.002–0.485
Basophils (*n*) (BASO#)	0.020	×10^9^/L	0–0.097

**Table 2:** Local hospital blood coagulation function test of Case 1.

Test name	Result	Unit	Reference range
Prothrombin time (PT)	11.2	Second	9–14.0
International normalized ratio (INR)	1.01		0.81–1.26
Activated partial thromboplastin time (APTT)	27.2	Second	20.0–40.0
Thrombin time (TT)	18.1	Second	13.0–24.0
Thrombin time ratio (TTR)	0.99		0.80–1.18
Fibrinogen (FG)	2.19	g/L	2–4

The patient was always healthy. A detailed medical history provided no information regarding abnormalities like fever, spontaneous bleeding, petechiae, or ecchymosis, except for recent slight fatigue. Clinical examination revealed no enlarged lymph nodes or other abnormalities. Oral hygiene was generally satisfactory, with only a small amount of plaque. Diffuse hyperplasia and swelling were observed in the buccal and lingual/palatal gingivae (Figure 1), extending to the attached gingiva. The gingival tissue appeared tender and edematous with positive bleeding on probing; however, no periodontal pockets were detected. No significant mobility was observed in any tooth. Panoramic radiography revealed slight alveolar bone absorption (Figure 2). The patient′s gingival swelling was inconsistent with the amount of plaque and radiographic results. To establish a definitive diagnosis, a localized gingival biopsy was required. Before doing so, hematologic disorders must be ruled out.

**Figure 1:**
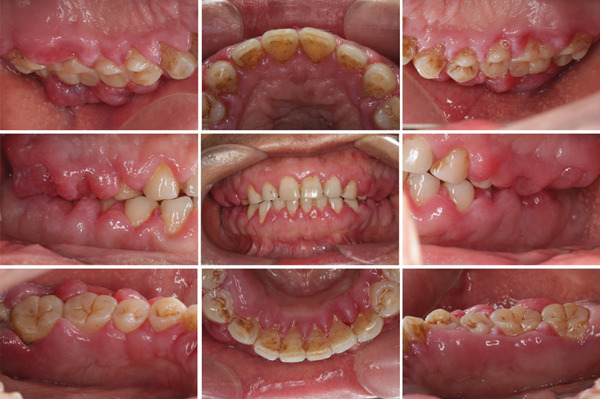
Intraoral images obtained at the first visit. Clinical examination shows gingival swelling, tenderness, and edema.

**Figure 2:**
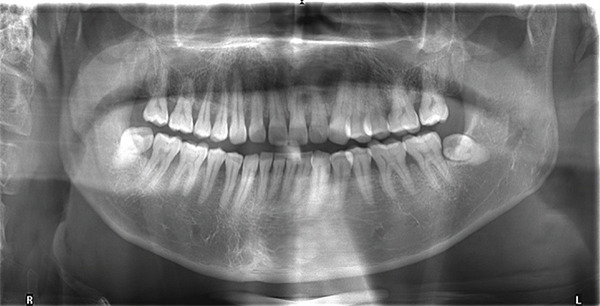
Radiographic image obtained at the first visit. The panoramic radiograph shows slight alveolar bone resorption.

Therefore, the patient was advised to undergo hematological testing again. The blood test results changed dramatically (Table 3). The patient was referred to the Department of Hematology.

**Table 3:** Complete blood count (CBC) at presentation of Case 1.

Test name	Result	Unit	Reference range
White blood cells (WBCs)	4.36	×10^9^/L	3.5–9.5
Red blood cells (RBCs)	4.23↓	×10^12^/L	4.3–5.8
Hemoglobin (HGB)	131.00	g/L	130–175
Hematocrit (HCT)	37.30↓	%	40–50
Mean corpuscular volume (MCV)	88.20	fL	82–100
Mean corpuscular hemoglobin (MCH)	31.00	pg	27–34
Red blood cell distribution width standard deviation (RDW‐SD)	43.60	fL	36.37–47.86
Red blood cell distribution width coefficient of variation (RDW‐CV)	14.30	%	12.2–14.8
Mean corpuscular hemoglobin concentration (MCHC)	351.00	g/L	316–354
Platelets (PLTs)	69.00↓	×10^9^/L	100–30
Neutrophils (NEUT%)	5.0↓	%	40–75
Lymphocytes (LYMPH%)	55.20↑	%	20–50
Monocytes (MONO%)	38.4	%	3–10
Eosinophils (EO%)	0.90	%	0.4–8.0
Basophils (BASO%)	0.5	%	≤ 1
Neutrophils (NEUT#)	0.21↓	×10^9^/L	1.8–6.3
Lymphocytes (LYMPH#)	2.34	×10^9^/L	1.1–3.2
Monocytes (MONO#)	1.63↑	×10^9^/L	0.01–0.6
Eosinophils (EO#)	0.04	×10^9^/L	0.02–0.52
Basophils (BASO#)	0.02	×10^9^/L	0–0.06

The hematologist immediately performed a bone marrow puncture. Bone marrow characteristics indicated hyperactive myelodysplasia, with sheets of blast cells (accounting for 81% of all). The cell sizes were variable and presented as round or oval. The cytoplasm was scant and pale blue, with some cells showing fine azurophilic granules (Figure 3). The nuclei were mostly quasicircular or oval, with visible indentations and folds. The proliferation of granulocytes and erythrocytes was suppressed, and no megakaryocytes were observed. Platelets were scattered, and degenerated cells were easily found. There were a lot of blasts, with a low distribution of white blood cells.

**Figure 3:**
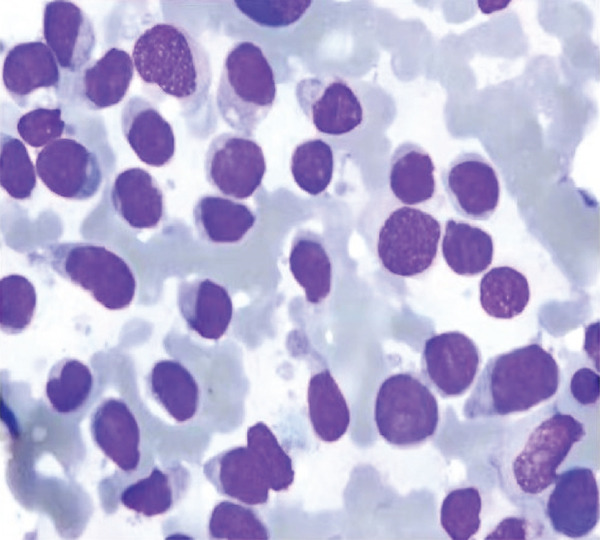
The images of bone marrow puncture (hematoxylin and eosin stain).

Flow cytometric analysis of peripheral blood demonstrated that primitive region cells (R3) accounted for 89.26% of all karyocytes, positive markers (CD13, CD15dim, CD33, CD34, CD38dim, CD56dim, CD64, CD117, and CD11C), and negative markers (CD5, CD7, CD9, CD10, CD16, CD19, CD36, CD123, HLA‐DR, and CD11B) (Figure 4). Granulocytes (R4) accounted for 1.84% of all karyocytes, and the majority were mature neutrophils. Based on these findings, acute myeloid leukemia (AML) was considered.

**Figure 4:**
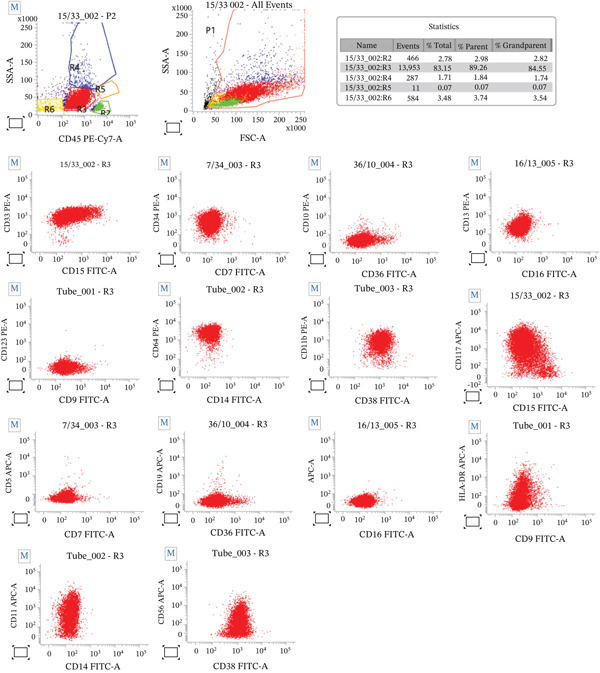
Flow cytometry of bone marrow analysis.

The “idarubicin plus cytarabine” chemotherapy regimen was administered. Meanwhile, oral hygiene instructions were provided to maintain oral health. One month after chemotherapy, the hematologist reported that gingival swelling had subsided. However, the patient′s condition unfortunately worsened after chemotherapy, and he eventually died.

### 2.2. Case 2

A 10‐year‐old boy consulted a dentist at the Department of Periodontics, the West China Hospital of Stomatology, Sichuan University, complaining of gingival pain and tooth mobility for 1 month. He was always healthy, and no abnormal findings like fever, bleeding, petechiae, ecchymosis, or dizziness were reported.

Intraoral examination revealed mixed dentition. Only small amounts of supragingival plaque and calculus were observed on the tooth surfaces. The gingiva appeared pink and was healthy. No periodontal pockets or dental caries was observed. However, all the teeth had Grade II mobility and pain on percussion (Figure 5). Clinical examination revealed no enlarged lymph nodes or other abnormalities. The patient had slight facial swelling, but pitting edema was not detected. No notable extraoral clinical findings were observed.

**Figure 5:**
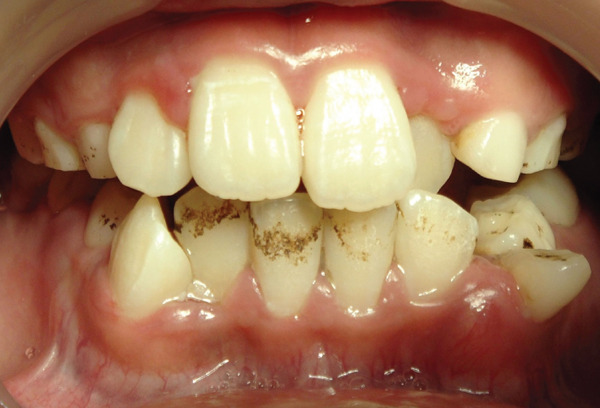
Intraoral images obtained at the first visit. Only small amounts of supragingival plaque and calculus are seen on the tooth surfaces. The gingiva is pink and healthy.

Panoramic computed tomography revealed no significant alveolar bone resorption. However, there was obvious bony crypt destruction around Teeth 13, 15, 17, 23, 24, 25, 27, 34, 35, 37, 44, 45, and 47, and the lamina dura was absent in Teeth 16, 26, 36, and 46. These osseous alterations were found in both the maxilla and mandible; however, the radiographic definition of the structures in the maxilla was inferior. The apical portions of Teeth 37 and 47 showed resorption with blurred radiolucent regions, whereas the apical lesions of Teeth 36 and 46 were blurred with obliteration of the root canals. The two developing lower third molars were almost completely destroyed, and Tooth 48 was displaced distally and occlusally. Additionally, the cortical outlines of the bilateral inferior alveolar canals had lost clarity (Figure 6). The patient was advised to undergo blood tests. However, neither the complete blood count (Table 4) nor coagulation function test results were remarkable (Table 5).

**Figure 6:**
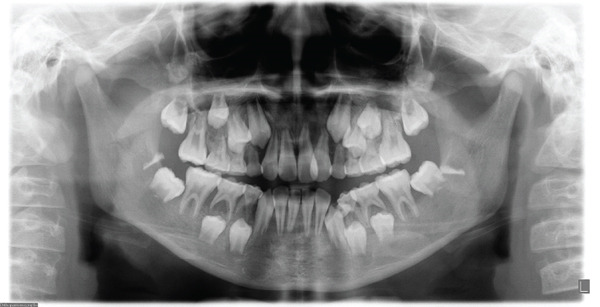
Radiographic image obtained at the first visit. The panoramic radiograph shows obvious bony crypt destructions around the developing teeth and the absence of erupted teeth. The apical portions of Teeth 37 and 47 are resorbed with blurred radiolucent regions, and the apical lesions of Teeth 36 and 46 are blurred with obliteration of root canals. The two developing lower third molars are displaced distally and occlusally. The cortical outline of bilateral inferior alveolar canals has also lost its clarity.

**Table 4:** Complete blood count (CBC) at presentation of Case 2.

Test name	Result	Unit	Reference range
White blood cells (WBCs)	11.48	×10^9^/L	5–12 (child)
Red blood cells (RBCs)	5.56↑	×10^12^/L	3.5–5.5
Hemoglobin (HGB)	146.00	g/L	110–160
Hematocrit (HCT)	44.90	%	35–50
Mean corpuscular volume (MCV)	80.80	fL	82–95
Mean corpuscular hemoglobin (MCH)	26.30	pg	27–32
Mean corpuscular hemoglobin concentration (MCHC)	325.00	g/L	320–360
Platelets (PLTs)	157.00	×10^9^/L	95–300
Mean platelet volume (MPV)	14.3↑	fL	9.4–12.5
Platelet larger cell ratio (P‐LCR)	55.80↑	%	13–43
Neutrophils (NEUT%)	64.84	%	50–70
Lymphocytes (LYMPH%)	24.74	%	20–40
Monocytes (MONO%)	8.04	%	3–10
Eosinophils (EO%)	1.34	%	0.5–5
Basophils (BASO%)	0.24	%	0–1
Neutrophils (*n*) (NEUT#)	7.44↑	×10^9^/L	1.6–6.8
Lymphocytes (*n*) (LYMPH#)	2.95	×10^9^/L	0.64–3.88
Monocytes (*n*) (MONO#)	0.920↑	×10^9^/L	0.096–0.776
Eosinophils (*n*) (EO#)	0.150	×10^9^/L	0.002–0.485
Basophils (*n*) (BASO#)	0.020	×10^9^/L	0–0.097

**Table 5:** Blood coagulation function test of Case 2.

Test name	Result	Unit	Reference range
Prothrombin time (PT)	15.4	Second	11.8–16.0
Prothrombin time ratio (PTR)	1.15		0.80–1.20
International normalized ratio (INR)	1.16↑		0.72–1.15
Fibrinogen (Fib)	6.57↑	g/L	1.69–5.15
Activated partial thromboplastin time (APTT)	28.1	Second	25.4–38.4
Activated partial thromboplastin time ratio (APTTR)	0.92		0.89–1.24
Thrombin time (TT)	14.3	Second	12.0–19.0
Thrombin time ratio (TTR)	0.99		0.80–1.18

Two days later, he complained of left thigh and shoulder pain, which he did not pay attention to. The patient was referred to West China Women′s and Children′s Hospital, Sichuan University, for further examination, but the boy′s parents neglected to do so. He did not undergo any examination until 40 days later. The patient received some therapy for a cold for several days, but the condition gradually deteriorated. He developed symptoms of acute nephritis and was immediately transferred to the West China Women′s and Children′s Hospital, Sichuan University, for further treatment. Complete blood counts and bone marrow puncture results confirmed the diagnosis of acute lymphoblastic leukemia (ALL; FAB L3, immunophenotype mature B‐ALL). At this point, his peripheral complete blood counts remained normal (white blood cell count, 7.3 × 10^9^/L; red blood cell count, 2.26 × 10^12^/L; hemoglobin, 61 g/L; and platelet count, 134 × 10^9^/L).

## 3. Discussion

Leukemia is a heterogeneous malignancy of the hematopoietic system, wherein tumor cells proliferate clonally in the bone marrow and blood [3]. It is a malignant disease with a high incidence in adolescents and adults [2], and the etiology of leukemia remains speculative. In 2019, approximately 21,450 new cases of AML were reported in the United States, leading to 10,920 deaths [4]; therefore, early diagnosis is important. Clinically, oral complications occur frequently in leukemia and may indicate initial evidence of the disease [5] or its relapse [6]. Oral lesions arise in both the acute and chronic forms of all types of leukemia and are far more frequent in the acute stages [7]. Oral manifestations of leukemia include gingival enlargement, gingival bleeding, oral ulcerations, petechiae, and mucosal pallor [8]. Complete blood count with differential is the preferred diagnostic test if the clinician suspects leukemia. However, in some cases, the oral manifestations of AL may precede systemic syndromes or routine blood indices.

In the two cases reported herein, unusual changes in the periodontal tissue, abnormal gingival enlargement, unusual gingival pain, and bone resorption on radiography were the initial symptoms with normal routine blood indices. Leukemia‐related gingival lesions are manifestations of malignant systemic diseases in localized areas. Although they present with common symptoms such as gum bleeding and hypertrophy, they differ from common gingival enlargement caused by local inflammation, drugs, gingival fibromatoses, or IgG4‐related gingival hyperplasia, exhibiting distinct characteristics.

Inflammation‐induced gingival enlargement is usually caused by hyperplastic gingivitis or periodontitis, with deposition of dental plaque and calculus. The associated gingival inflammation is accompanied by edema, bleeding, swelling, or generalized gingival enlargement. Typically, the lesions appear red and friable and bleed easily. Once the plaque and calculus are removed, the gingival enlargement should improve.

Drug‐induced hyperplasia is associated with specific drugs, including antihypertensive drugs (dipine drugs), calcineurin inhibitors (cyclosporine), and antiseizure drugs (phenytoin). These medications can affect all gingiva sites, from the free gingiva to the attached gingiva. When the gingival hyperplasia is accompanied by inflammation, the gingiva appears red and friable and bleeds easily. Once periodontal inflammation is relieved and oral hygiene is improved, the gingiva becomes pink, firm, and painless.

IgG4‐related gingival hyperplasia presents as a diffuse swelling of the gums, with moderate congestion and redness at the edges, soft texture, and elasticity affecting the attached gums. It is characterized by elevated serum IgG4 levels and extensive infiltration of IgG4‐positive plasma cells into the gum tissue. Most patients with IgG4 syndrome exhibit systemic manifestations. The diagnosis of IgG4‐related disease primarily relies on the following points: characteristic diffuse/localized swelling or mass in one or more organs, elevated serum IgG4 concentration, and dense lymphoplasmacytic infiltration, storiform fibrosis, and obliterative phlebitis with abundant IgG4+ plasma cells in the affected tissue [9].

In gingival fibromatoses, gingival enlargement usually occurs during childhood and coincides with the eruption of primary or permanent dentition. Widespread gradual gingival hyperplasia may involve the gingival margin, interdental papillae, and attached gingiva of the entire dentition, sometimes extending to the mucogingival junction. The hyperplastic gingiva is firm and resilient, with little tendency to bleed. It may cover a portion or the entire tooth crown, thereby interfering with mastication. Due to gingival thickening, delayed tooth eruption may occasionally occur. The condition may manifest either sporadically or in association with various syndromes, including Hunter syndrome and prune belly syndrome [10].

The above are relatively common diseases that present with diffuse gingival hyperplasia. There are also some rarer diseases, like spongiotic gingival hyperplasia and granulomatosis with polyangiitis. The diagnosis of these diseases depends on the medical history, biopsy, and histopathologic examination (Table 6).

**Table 6:** Differential diagnosis of gingival enlargement in different diseases.

Diseases	Medical/drug history	Clinical oral manifestations	Pathological finds	Diagnostic criteria
Hyperplastic gingivitis/periodontitis	Noncontributory	1. Poor oral hygiene 2. Gingival edema, bleeding, swelling, or generalized	Nonpathognomonic	Clinical and radiographic evaluation
Drug‐induced gingival enlargements	1. Antihypertensive drug 2. Calcineurin inhibitor drug 3. Antiseizure drug	1. Gingival hyperplasia may involve all gingival sites 2. The gingiva is pink, firm, and painless when no periodontal inflammation	Nonpathognomonic	Clinical, radiographic evaluation and medical history
IgG4‐related gingival hyperplasia	Noncontributory	A diffuse swelling, moderate congestion, redness, and soft texture	Dense lymphoplasmacytic infiltration, storiform fibrosis, and obliterative phlebitis with abundant IgG4+ plasma cells	1. Diffuse/localized swelling or mass in one or more organs 2. Elevated serum IgG4 concentration 3. Dense lymphoplasmacytic infiltration, storiform fibrosis, and obliterative phlebitis with abundant IgG4+ plasma cells
Gingival fibromatosis	Noncontributory	1. Widespread gradual gingival hyperplasia 2. Firm and resilient, little tendency to bleed 3. Delayed tooth eruption	1. Excess collagen 2. Enlarged fibroblasts 3. Coarse and fine dense collagen fiber bundles [11]	1. Hereditary, onset in childhood 2. Clinical, radiographic evaluation and medical history
Spongiotic gingival hyperplasia	Noncontributory	Red sessile lesions with a velvet or pebbly surface and soft consistency	1. Inflammatory infiltration of the lamina propria (lymphocytes and plasma cells) 2. Abundant vascularization in connective tissue [12]	Clinicopathologic correlation, IHC evaluation (CK19 and CK8/18)
Granulomatosis with polyangiitis	Noncontributory	“Strawberry‐like” gingivitis	Laboratory tests (including PR3‐ANCA or c‐ANCA)	1. Sinonasal sign 2. Auditory function 3. PR3‐ANCA or c‐ANCA 4. Radiographic evaluation (chest) 5. Renal and respiratory tract involvement [13]
Leukemia	Noncontributory	Gingival enlargement, gingival bleeding, oral ulcerations, petechiae, and mucosal pallor	Atypical cells with myelomonocytic or lymphoid characteristics (biopsy is contraindicated during the acute phase of leukemia)	Bone marrow aspiration

Gingival hyperplasia in AL, as part of early oral signs and symptoms caused by leukemic infiltration or an exaggerated secondary periodontal inflammatory response or both, comprises 5% of the early complications [14]. It was the most prevalent clinical oral finding of AL in the available investigations [15]. Gingival hyperplasia is characterized by the progressive proliferation of fibrous connective tissue, causing enlargement of the interdental papillae and the marginal and attached gingiva, slowly covering the crowns of the teeth [16]. Approximately 70%–80% of patients with de novo AML achieve complete remission following standard chemotherapy [17].

The gingiva in the second case had a normal appearance without obvious periodontal inflammation, but the chief complaint was gingival pain and loosening of teeth. The patient′s general condition and blood test results were unremarkable. However, panoramic radiography showed destruction or the absence of bony crypts around the developing teeth and the lamina dura of the erupted teeth. Osseous changes in the jaws of patients with leukemia have been reported previously. Curtis [18] reviewed 214 panoramic dental radiographs of children with AL and found loss or thinning of the crypts of developing teeth and loss of the lamina dura of erupted teeth. This was similar to that observed in our patient. The researchers found that these osseous changes were observed in 62.9% of children with active leukemia (including the relapse of leukemia) and the earliest manifestations were usually seen in the molar regions and the apical part of the alveolar bone [18]. Benson et al. [6] described a case of mandibular involvement in the relapse of childhood ALL. Radiographic images showed similar destruction of the jaw, as observed in our case. Other bone changes in the jaw have been reported. Bakathir and Al‐Hamdani [19] and Bernstein and Schwartz [20] demonstrated that the apical portion of some teeth was destroyed with areas of ill‐defined radiolucency in radiographic images of the jaw in a patient with relapse of ALL and the loss of the bilateral inferior alveolar canal also disappeared. These pathological changes were observed in the present case. Therefore, the unusual radiological appearance of the mandible might suggest leukemic infiltration, and further medical investigation is urgently required.

## 4. Recommendation

Although physicians most commonly diagnose leukemia, they focus more on AL treatment. Moreover, the diagnosis of AL by physicians is mainly based on bone marrow puncture accompanied by an abnormal complete blood count, and they have no chance to explore whether there are other clinical symptoms, while routine blood tests show no abnormalities. Oral manifestations, especially in periodontal tissues, are often the first indication of leukemia. Therefore, dentists are responsible for the initial diagnosis of AL and the race against time for subsequent therapy. Dentists must be adept at observing subtle differences in common symptoms during clinical practice, recognizing the intrinsic connections between various localized symptoms, linking these local manifestations to hematological diseases, and instructing patients to undergo further examinations. Dentists should be aware of the diagnostic signs and complications associated with leukemia and offer insights into the diagnosis of rare diseases.

## Author Contributions

Jingmei Yang: conceptualization, investigation, methodology, supervision, validation, visualization, writing—original draft, and writing—review and editing; Wenwen Chen: data curation, resources, investigation, validation, visualization, and writing—original draft; Panpan Wang: data curation, resources, visualization, and writing—original draft; Jiajia Liu: formal analysis, funding acquisition, visualization, and writing—original draft; Yi Xu: conceptualization, formal analysis, investigation, methodology, supervision, validation, visualization, writing—original draft, and writing—review and editing. Jingmei Yang and Wenwen Chen contributed equally to this work.

## Funding

This research was funded by the National Natural Science Foundation of China (82401131).

## Disclosure

All authors have read and approved the final version of the manuscript. The corresponding authors, who also serve as the manuscript guarantors, had full access to all of the data in this study and take complete responsibility for the integrity of the data and the accuracy of the data analysis.

## Ethics Statement

The authors have nothing to report.

## Consent

The patients′ family in these two cases gave their informed consent for the case publication.

## Conflicts of Interest

The authors declare no conflicts of interest.

## Data Availability

The data that support the findings of this study are available from the corresponding authors upon reasonable request.

## References

[1] Sinrod H. S. , Leukemia as a Dental Problem, Journal of the American Dental Association. (1957) 55, no. 6, 809–818, 10.14219/jada.archive.1957.0251. 13480784

[2] Bray F. , Laversanne M. , Sung H. , Ferlay J. , Siegel R. L. , Soerjomataram I. , and Jemal A. , Global Cancer Statistics 2022: GLOBOCAN Estimates of Incidence and Mortality Worldwide for 36 Cancers in 185 Countries, CA. a Cancer Journal for Clinicians. (2024) 74, no. 3, 229–263, 10.3322/caac.21834, 38572751. 38572751

[3] Kowalczyk A. , Zarychta J. , Lejman M. , and Zawitkowska J. , Electrochemical and Optical Sensors for the Detection of Chemical Carcinogens Causing Leukemia, Sensors. (2023) 23, no. 7, 10.3390/s23073369. PMC10098728 37050429

[4] Pandya B. J. , Chen C. C. , Medeiros B. C. , McGuiness C. B. , Wilson S. D. , Walsh E. H. , and Wade R. L. , Economic and Clinical Burden of Acute Myeloid Leukemia Episodes of Care in the United States: A Retrospective Analysis of a Commercial Payer Database, Journal of Managed Care & Specialty Pharmacy. (2020) 26, no. 7, 849–859, 10.18553/jmcp.2020.19220, 32281456. 32281456 PMC10391266

[5] Aronovich S. and Connolly T. W. , Pericoronitis as an Initial Manifestation of Acute Lymphoblastic Leukemia: A Case Report, Journal of Oral and Maxillofacial Surgery. (2008) 66, no. 4, 804–808, 10.1016/j.joms.2006.10.062, 18355610. 18355610

[6] Benson R. E. , Rodd H. D. , North S. , Loescher A. R. , Farthing P. M. , and Payne M. , Leukaemic Infiltration of the Mandible in a Young Girl, Journal of Oral and Maxillofacial Surgery. (2007) 17, no. 2, 145–150, 10.1111/j.1365-263X.2006.00794.x, 17263867. 17263867

[7] Stafford R. , Sonis S. , Lockhart P. , and Sonis A. , Oral Pathoses as Diagnostic Indicators in Leukemia, Oral Surgery, Oral Medicine, Oral Pathology. (1980) 50, no. 2, 134–139, 10.1016/0030-4220(80)90200-5, 6967202. 6967202

[8] Francisconi C. F. , Caldas R. J. , Oliveira Martins L. J. , Fischer Rubira C. M. , and da Silva Santos P. S. , Leukemic Oral Manifestations and Their Management, Asian Pacific Journal of Cancer Prevention. (2016) 17, no. 3, 911–915, 10.7314/APJCP.2016.17.3.911, 27039811. 27039811

[9] Wallace Z. S. , Naden R. P. , Chari S. , Choi H. K. , Della-Torre E. , Dicaire J. F. , Hart P. A. , Inoue D. , Kawano M. , Khosroshahi A. , Lanzillotta M. , Okazaki K. , Perugino C. A. , Sharma A. , Saeki T. , Schleinitz N. , Takahashi N. , Umehara H. , Zen Y. , Stone J. H. , and Members of the ACR/EULAR IgG4-RD Classification Criteria Working Group , The 2019 American College of Rheumatology/European League Against Rheumatism Classification Criteria for IgG4-Related Disease, Annals of the Rheumatic Diseases. (2020) 79, no. 1, 77–87, 10.1136/annrheumdis-2019-216561, 31796497. 31796497

[10] Papadopoulou E. , Kouri M. , Andreou A. , Diamanti S. , Georgaki M. , Katoumas K. , Damaskos S. , Vardas E. , Piperi E. , and Nikitakis N. G. , Challenges in Differential Diagnosis of Diffuse Gingival Enlargement: Report of Two Representative Cases and Literature Review, Dentistry Journal. (2024) 12, no. 12, 10.3390/dj12120403, 39727460. PMC11674662 39727460

[11] Gawron K. , Łazarz-Bartyzel K. , Potempa J. , and Chomyszyn-Gajewska M. , Gingival Fibromatosis: Clinical, Molecular and Therapeutic Issues, Orphanet Journal of Rare Diseases. (2016) 11, no. 1, 10.1186/s13023-016-0395-1. PMC4729029 26818898

[12] Allon I. , Lammert K. M. , Iwase R. , Spears R. , Wright J. M. , and Naidu A. , Localized Juvenile Spongiotic Gingival Hyperplasia Possibly Originates From the Junctional Gingival Epithelium-An Immunohistochemical Study, Histopathology. (2016) 68, no. 4, 549–555, 10.1111/his.12774, 26154781. 26154781

[13] Robson J. C. , Grayson P. C. , Ponte C. , Suppiah R. , Craven A. , Judge A. , Khalid S. , Hutchings A. , Watts R. A. , Merkel P. A. , Luqmani R. A. , and for the DCVAS Study Group , 2022 American College of Rheumatology/European Alliance of Associations for Rheumatology Classification Criteria for Granulomatosis With Polyangiitis, Arthritis and Rheumatology. (2022) 74, no. 3, 393–399, 10.1002/art.41986. 35106964

[14] Willams W. J. , Hematology, McGraw Hill.

[15] Busjan R. , Hasenkamp J. , Schmalz G. , Haak R. , Trümper L. , and Ziebolz D. , Oral Health Status in Adult Patients With Newly Diagnosed Acute Leukemia, Clinical Oral Investigations. (2018) 22, no. 1, 411–418, 10.1007/s00784-017-2127-x, 28536781. 28536781

[16] Sepúlveda E. , Brethauer U. , Fernández E. , Cortés G. , and Mardones C. , Oral Manifestations as First Clinical Sign of Acute Myeloid Leukemia: Report of a Case, Pediatric Dentistry. (2012) 34, no. 5, 418–421, 23211920. 23211920

[17] Löwenberg B. , Downing J. R. , and Burnett A. , Acute Myeloid Leukemia, New England Journal of Medicine. (1999) 341, no. 14, 1051–1062, 10.1056/NEJM199909303411407. 10502596

[18] Curtis A. B. , Childhood Leukemias: Osseous Changes in Jaws on Panoramic Dental Radiographs, Journal of the American Dental Association. (1971) 83, no. 4, 844–847, 10.14219/jada.archive.1971.0405. 5289210

[19] Bakathir A. A. and Al-Hamdani A. S. , Relapse of Acute Lymphoblastic Leukemia in the Jaw, Oral Surgery, Oral Medicine, Oral Pathology, Oral Radiology, and Endodontology. (2009) 107, no. 5, e14–e16. 10.1016/j.tripleo.2009.01.029 19426901

[20] Bernstein M. L. and Schwartz S. , Mandibular Relapse in Acute Leukemia. Report of a Case, American Journal of Pediatric Hematology Oncology. (1986) 8, no. 3, 240–244. 3490189 10.1097/00043426-198623000-00012

